# Right data for right patient—a precisionFDA NCI–CPTAC Multi-omics Mislabeling Challenge

**DOI:** 10.1038/s41591-018-0180-x

**Published:** 2018-09-07

**Authors:** Emily Boja, Živana Težak, Bing Zhang, Pei Wang, Elaine Johanson, Denise Hinton, Henry Rodriguez

**Affiliations:** 1 Office of Cancer Clinical Proteomics Research, Center for Strategic Scientific Initiatives, National Cancer Institute, National Institutes of Health, Bethesda, MD USA; 2 Office of In Vitro Diagnostics and Radiological Health, Center for Devices and Radiological Health, US Food and Drug Administration, Silver Spring, MD USA; 3 Department of Molecular and Human Genetics, Baylor College of Medicine, Houston, TX USA; 4 Department of Genetics and Genomic Sciences, Icahn School of Medicine at Mount Sinai, New York, NY USA; 5 Office of Health Informatics, Office of the Chief Scientist, Office of the Commissioner, US Food and Drug Administration, Silver Spring, MD USA; 6 Office of the Chief Scientist, Office of the Commissioner, US Food and Drug Administration, Silver Spring, MD USA

**Keywords:** Bioinformatics, Medical genomics

## Abstract

To address a critical roadblock that can occur in translational and clinical research, the National Cancer Institute and the Food and Drug Administration, in coordination with the DREAM Challenges, are launching the first computational challenge using multi-omics datasets to detect and correct specimen mislabeling.

Although genomics has shaped the current scope of precision medicine, it is becoming increasingly clear that molecular phenotypes, such as DNA and RNA profiles and, in particular, protein abundance profiles, are essential to our understanding of biology and for enhancing our ability to achieve the promise of precision medicine for patients. Hence, simultaneous generation and integration of multidimensional multi-omics datasets from a large set of tumor samples, such as those used in the National Cancer Institute’s (NCI) The Cancer Genome Atlas (TCGA; https://cancergenome.nih.gov) and the Clinical Proteomic Tumor Analysis Consortium (CPTAC; https://proteomics.cancer.gov) projects^1–4^, is becoming a powerful approach to understanding the molecular basis of diseases and speeding the translation of new discoveries to patient care. This development has been largely enabled by the rapid technological advancement, standardization and harmonization in tumor molecular profiling in recent years. Consequently, several initiatives have been launched to leverage this development for application to clinical practice, including the International Cancer Proteogenome Consortium^5^ and the Applied Proteogenomics Organizational Learning and Outcomes^6^ programs. These efforts promise to revolutionize our understanding of cancer biology and change the way cancer is treated.

The value of multi-omics technologies and datasets lies in the possibility of accurately extracting rich information to help understand the molecular complexities specific to individual patients through use of sophisticated integrative computational algorithms. Such information can be used to reach a deeper understanding of a disease, which then can be applied clinically, for example, to elucidate the relationship between the genome and proteome of a patient’s tumor or to deconvolute tumor heterogeneity associated with clinical outcome. Ideally, individual and population data would ultimately serve to inform a physician and a patient and to help determine the most appropriate treatment options. Furthermore, the comprehensive information obtained on the same sample in multiple dimensions can add value in pinpointing and correcting problems that can be encountered, such as sample mislabeling by accidental swapping of patient samples or data mislabeling (accidental swapping of patient omics data), which could lead to multiple patients receiving the wrong medical treatment, resulting in severe, irreversible consequences.

Sample mislabeling that contributes to irreproducible results and invalid conclusions is known to be one of the obstacles in basic and translational research^7^. This is also prevalent in data-rich large-scale omics studies^8,9^, in which human errors could arise anywhere in the data production and analysis pipeline—either sample mislabeling (early in the pipeline) or data mislabeling (later in the pipeline).

The Food and Drug Administration (FDA) and NCI-CPTAC, with a history of collaboration^10^, also have experience in building challenges, such as the precisionFDA Challenges (https://precision.fda.gov/challenges) and NCI–CPTAC DREAM Proteogenomics Challenge (https://www.synapse.org/#!Synapse:syn8228304/wiki/413428), to solve complex problems. Now they are joining forces to launch a Multi-omics Enabled Sample Mislabeling and Correction Challenge (https://precision.fda.gov/mislabeling) in September 2018. The objective of this challenge is to encourage development and evaluation of computational algorithms that can accurately detect and correct mislabeled samples using rich multi-omics datasets, enhancing the assurance that the right data is attributed to the right patient.

## Challenge design

The challenge comprises two subchallenges to be conducted sequentially. In Subchallenge 1, participants will be asked to detect mislabeled samples. Participants will be presented with a training dataset and a test dataset, comprising real-world clinical and proteomics data. Mislabeled samples will be known in the training dataset and not known in the test dataset. Using the training dataset, participants will develop computational models to distinguish samples of matched and nonmatched clinical and proteomics data. The computational models will then be used to identify mislabeled samples in the test dataset.

**Fig. 1:**
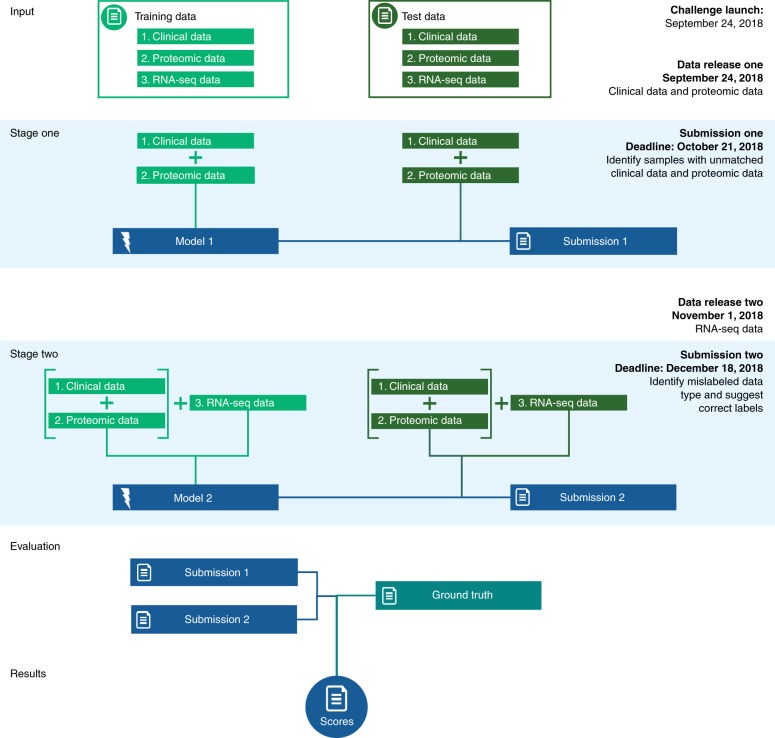
Challenge design and timelines.

In Subchallenge 2, participants will be asked to correct mislabeled samples in richer data. Participants will be presented with real-world RNA profiling data for all samples in both the training and test datasets. Similar to the clinical and proteomics data, newly introduced RNA profiling data will also include mislabeled samples. As with Subchallenge 1, this information will be known in the training dataset, but not in the test dataset. Participants will develop computational algorithms to model the relationships among the three data types in the training dataset and then will apply the computational model to identify and correct instances of single data type sample mislabeling among the trio of data types in the test dataset. Subchallenge results will be independently evaluated (Fig. 1).

## Anticipated outcome and impact

An immediate outcome envisioned is a flagship challenge manuscript that gives an overview of the challenge data, questions, design, and outcomes^11^. Additionally, the algorithms that the participants propose will be aggregated with the aim of refining a final open-source product to be incorporated into an analysis pipeline and ultimately as part of a quality-management system to reduce errors. This could help speed the translation of multidimensional omics technologies and datasets to the clinic. Meanwhile, NCI and FDA hope to build and expand a community of scientists that will collaborate to solve important problems that prevent the translation of multi-omics data to the clinical labs.
